# P-886. Small Intestinal Bacterial Overgrowth and Bloodstream Infection in the Pediatric Intestinal Failure Population

**DOI:** 10.1093/ofid/ofae631.1077

**Published:** 2025-01-29

**Authors:** Tessa Barclay, Melissa Campbell, Ibukun Kalu, Debra Sudan

**Affiliations:** Duke University Medical Center, Durham, North Carolina; Duke University Medical Center, Durham, North Carolina; Duke University, Durham, NC; Duke University, Durham, NC

## Abstract

**Background:**

Patients with intestinal failure (IF) are reliant on central venous access for adequate nutrition, and thus are at risk of catheter-related infectious complications, including bloodstream infection (BSI) (1). These patients may suffer from small intestinal bacterial overgrowth (SIBO), with symptoms including abdominal bloating and diarrhea. SIBO can lead to poor enteral feeding tolerance and malnutrition (2). SIBO diagnosis is made via jejunal aspirate, hydrogen breath testing, or clinical suspicion based on relevant symptoms, and is often treated with prolonged antibiotics (2). There is a paucity of data investigating the effect of prolonged antibiotic exposure from SIBO on BSI episodes in this population. This study seeks to elucidate the connection between SIBO-related antibiotic exposure, frequency of BSI episodes, and antimicrobial resistance patterns of the causative organisms in the pediatric IF population.

**Table 1:**
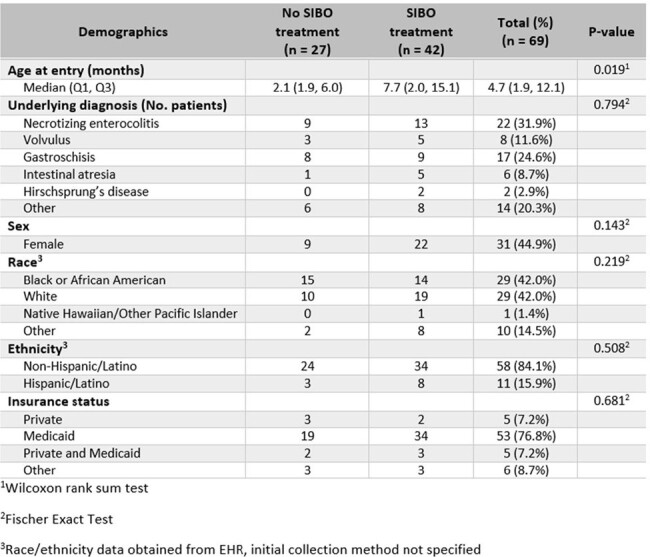
Demographics of Pediatric Intestinal Failure Cohort (2009 – 2023)

Sixty-nine patients met inclusion criteria for this study. Intestinal failure was defined as reliance on parenteral nutrition for ≥ 60 days with an associated diagnosis of short bowel syndrome or other condition associated with intestinal failure. The majority of patients entered the cohort prior to one year of life (median age 4.7 months), a result which was statistically significant among patients ever treated for small intestinal bacterial overgrowth compared to those never treated. Necrotizing enterocolitis was the most common underlying diagnosis leading to intestinal failure.

**Methods:**

A retrospective chart review was conducted from January 2009 through June 2023 for patients treated in the Duke Pediatric Intestinal Rehabilitation Program. Data was stored within a RedCap database per institutional IRB protocol. Statistical analysis performed using SAS 9.4 (SAS Institute Inc., Cary, NC).

**Table 2:**
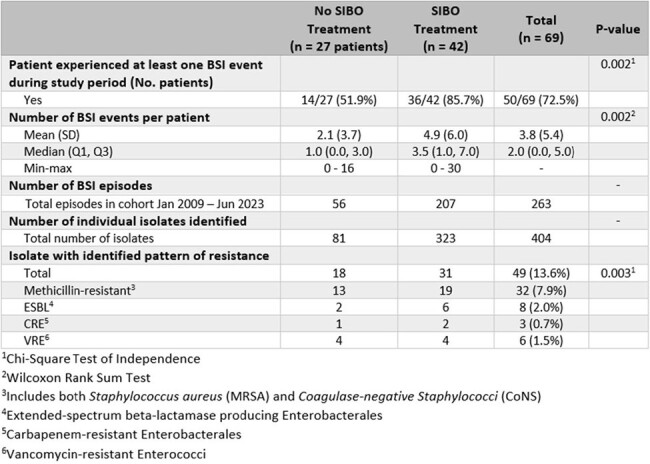
Description of BSI Episode Characteristics Among Patients Treated for Small Intestinal Bacterial Overgrowth Compared to No Treatment

The majority of patients (61%) in the study cohort received treatment for small intestinal bacterial overgrowth (SIBO) at any point. Patients treated for SIBO were more likely to experience a bloodstream infection (BSI) event, and on average had a higher number of BSI events compared to those never treated for SIBO. Patients treated for SIBO were also more likely to have a BSI event including an organism with an identified pattern of resistance. BSI events were defined per CDC/NHSN LCBI 1 criteria, excepting in the case of commensals.

**Results:**

The cohort included 69 patients, of whom 42 (61%) were treated for SIBO at any point during the study period. Median treatment length was 68 weeks (IQR 17.4 – 196.1). 73% of patients experienced a BSI event, with 263 episodes total. The median number of BSI episodes was higher for patients treated for SIBO at any point (p = 0.002). *Klebsiella pneumoniae* was the most commonly cultured organism (14% of isolates), followed by *Coagulase-negative Staphylococci* (13%). Thirteen percent of the isolates had an identified pattern of resistance, most commonly methicillin-resistance.

**Figure:**
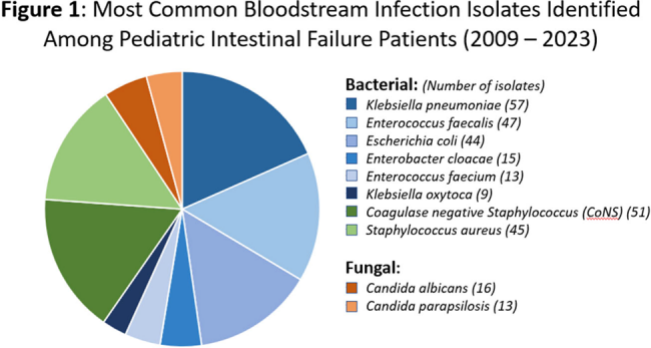

There was a total of 263 bloodstream infection (BSI) episodes in the cohort, with 404 individual isolates identified. Enteric isolates were most common, namely Klebsiella pneumoniae and Enterococcus faecalis.

**Conclusion:**

The majority of patients with IF received treatment for SIBO based on clinical diagnostic criteria. Treatment duration was prolonged, despite a lack of evidence for this practice. BSI was common in this cohort, most frequently with enteric pathogens. Preliminary analysis shows a higher median number of BSI episodes in patients ever treated for SIBO. Future applications of this data may include developing an institutional algorithm standardizing SIBO treatment.

**Disclosures:**

**All Authors**: No reported disclosures

